# Expression of PD-1/PD-L1 and PD-L2 in peripheral T-cells from non-small cell lung cancer patients

**DOI:** 10.18632/oncotarget.22025

**Published:** 2017-10-24

**Authors:** Oscar Arrieta, Edgar Montes-Servín, Juan-Manuel Hernandez-Martinez, Andrés F. Cardona, Eibar Casas-Ruiz, José C. Crispín, Daniel Motola, Diana Flores-Estrada, Lourdes Barrera

**Affiliations:** ^1^ Functional Unit of Thoracic Oncology and Laboratory of Personalized Medicine, Instituto Nacional de Cancerología, Mexico City, Mexico; ^2^ CONACyT-Instituto Nacional de Cancerología, Mexico City, Mexico; ^3^ Clinical and Translational Oncology Group, Institute of Oncology, Clínica del Country, Bogotá, Colombia; ^4^ Department of Immunology and Rheumatology, Instituto Nacional de Ciencias Médicas y Nutrición Salvador Zubirán, Mexico City, Mexico; ^5^ Centro Oncológico, Hospital Médica Sur, Mexico City, Mexico

**Keywords:** immunotherapy, checkpoint inhibitors, lung adenocarcinoma, prognosis, circulating lymphocytes

## Abstract

Binding of programmed death-1 (PD-1) with its ligands (PD-L1/2) transmits a co-inhibitory signal in activated T-cells that promotes T-cell exhaustion, leading to tumor immune evasion. The efficacy of antibodies targeting PD-1 and PD-L1 has led to a paradigm shift in lung cancer treatment but the prognostic and predictive value of tumor PD-L1 expression remains controversial. Evaluating PD-1, PD-L1/2 expression in peripheral blood cells may serve as a potential biomarker for prognosis and response to therapy. In this prospective observational study, plasma cytokine levels and PD-1, PD-L1 and PD-L2 expression was evaluated in circulating CD3^+^, CD3^+^CD4^+^ and CD3^+^CD8^+^ cells from 70 treatment-naïve patients with advanced NSCLC (Stage IIIB and IV) and from 10 healthy donors. The primary objective was to assess OS according to PD-1, PD-L1, PD-L2 expression status on PBMCs and lymphocyte subsets. Our results indicate that the percentage of PD-L1^+^CD3^+^, PD-L1^+^CD3^+^CD8^+^ PD-L2^+^PBMCs, PD-L2^+^CD3^+^, PD-L2^+^CD3^+^CD4^+^ cells was higher in patients than in healthy donors. Survival was decreased among patients with a high percentage of either PD-1^+^PBMCs, PD-1^+^CD3^+^, PD-L1^+^CD3^+^, PD-L1^+^CD3^+^CD8^+^, PD-L2^+^CD3^+^, PD-L2^+^CD3^+^CD4^+^, or PD-L2^+^CD3^+^CD8^+^ cells. IL-2 and TNF-α showed the strongest association with PD-L1 and PD-L2 expression on specific subsets of T-lymphocytes. Our findings suggest that increased PD-1/PD-L1/PDL-2 expression in PBMCs, particularly in T-cells, may be an additional mechanism leading to tumor escape from immune control. This study is registered with ClinicalTrials.gov, number NCT02758314.

## INTRODUCTION

Lung cancer remains the leading cause of cancer-related deaths in men, and had, in 2015, the second highest absolute incidence globally. It has been estimated that in 2017 lung cancer will account for 13% of all new cancer cases and for 26% of cancer-related deaths [1]. Non-Small Cell Lung Cancer (NSCLC) is the predominant type, accounting for approximately 85% of all newly diagnosed cases, with the majority of patients exhibiting advanced-stage disease [2, 3]. It is now widely accepted that the immune system plays a crucial role in preventing or promoting the development and progression of several types of cancer, including lung cancer. Immunotherapy has thus become an important treatment strategy whereby several components of the immune system are targeted to elicit effective anti-tumor responses. Immunotherapeutic approaches to lung cancer have shown great potential to improve treatment outcomes [4].

Transformed cells can evade immune system elimination by decreasing the expression of antigen presentation molecules and co-stimulatory molecules or, by increasing the expression of co-inhibitory molecules [5–8] such as programmed cell death protein 1 (PD-1). PD-1 is expressed on the surface of activated macrophages, T-lymphocytes, B lymphocytes, NK cells, and on some myeloid cells [9–12], where it inhibits the survival, proliferation and function through its interaction with PD-L1 and L2. The interaction of PD-1 with its ligands attenuates immune responses [13] and protects tumor cells from cytotoxic T-cell attack, leading to immune system evasion [14]. Immune checkpoint inhibitors (ICIs) are antibodies that target co-inhibitory molecules, such as PD-1/PD-L1, to improve anti-tumor immune responses [15, 16].

Anti-PD-1 ICIs have achieved higher therapeutic responses than standard-of-care chemotherapy in patients with advanced NSCLC, both in the second-line [17–19] and first-line [20] setting. In these studies, tumor PD-L1 expression was generally associated with higher responses to anti-PD-1 therapy [17, 18, 21–23]. However, a significant number of patients with PD-1/PD-L1 positive tumors fail to respond to treatment with anti-PD-1 ICIs and a significant number of patients (15-40%) with PD-L1 negative tumors benefit from anti-PD-1 ICIs. Consequently, there is still considerable debate with regards to the prognostic and predictive value of tumor PD-L1 expression in stratifying patients eligible for such interventions. Several reports have shown that the expression of PD-1/PD-L1/PD-L2 on the surface of infiltrating T-cells and circulating tumor cells (CTCs) is associated with poor prognosis [24, 25]. However, the prognostic value of PD-1/PD-L1/PD-L2 expression on peripheral T-cells from NSCLC patients has not been sufficiently evaluated. The aim of this study was to determine whether the expression of PD-1/PD-L1/PD-L2 on PBMCs, particularly on T-cell subsets, was associated with different survival outcomes in NSCLC patients.

## RESULTS

### Patient demographics

Supplementary Table 1 shows that patients (N=70) and healthy donors (N=10) exhibited an even distribution with regards to age, gender, diabetes and hypertension. The only significant difference between groups was with regards to smoking history which was reported by 54.3% of NSCLC patients. Among NSCLC patients, 32.8% had an EGFR mutation and 88.6% presented adenocarcinoma histology. At the time of diagnosis, 14.3% of patients presented stage IIIB diseases while the remaining 85.7% had stage IV disease. Brain metastases and pleural effusion was found in 35.7% and 68.6% of patients, respectively. Most patients (97.1%) presented an ECOG of 0-1. Approximately 93% of patients received different regimens of chemotherapy: 80% were treated with Platinum-Taxol; 4.3% with Pemetrexed; 2.9% Platinum-Gemcitabine; 5.7% with Platinum-Pemetrexed. Only 7.1% of patients with EGFR mutations received TKI as first-line treatment. All other patients with EGFR mutations received TKI as a second-line treatment due to delays in obtaining genetic profile results. Patient characteristics are summarized in Supplementary Table 1.

### PD-1/PD-L1/PD-L2 expression on T-cells from NSCLC patients

No differences were found between patients and controls with regards to the percentage of CD3^+^, CD3^+^CD8^+^, PD-1^+^PBMCs, PD-1^+^CD3^+^, PD-1^+^CD3^+^CD4^+^, PD-1^+^CD3^+^CD8^+^, PD-L1^+^CD3^+^CD4^+^ and PD-L2^+^CD3^+^CD8^+^ cells (*P*=0.7813, *P*=0.6631, *P*=0.810, *P*=0.9400, *P*=0.8550, *P*=7881, *P*=0.1213 and *P*=0.0812, respectively).

However, patients had a lower percentage of CD3^+^CD4^+^ cells than controls (46.07 ± 14.62 vs 61.46 ± 6.36; *P*=0.0016) as well as a lower percentage of PD-L1^+^PBMCs (0.77 [95% CI 0–4.62] vs 1.1410 [95% CI 0.71–2.90; *P*=0.0376).

**Figure 1:**
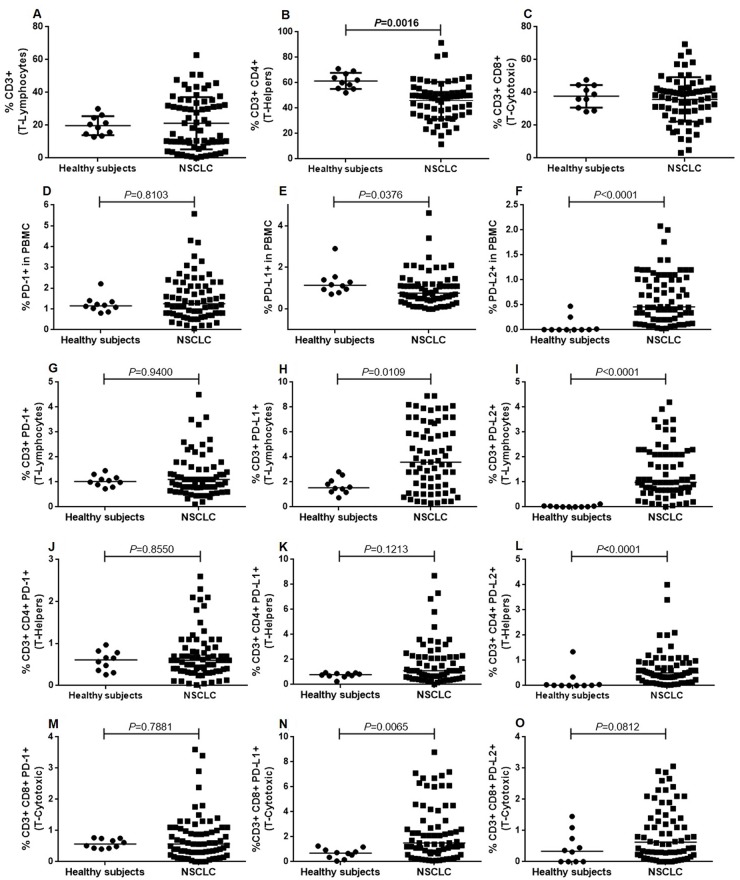
Immune cell subsets in NSCLC patients and healthy subjects Scatter plot showing the percentage of **(A)** CD3 +, **(B)** CD3 +CD4 +, **(C)** CD3 +CD8 + cells in the PBMC fraction. Panel **(D, E)** & **(F)** show the percentage of PBMCs positive for PD-1, PD-L1 & PD-L2, respectively. Panel **(G, H)** & **(I)** show the percentage of PD-1 +CD3 +, PD-L1 +CD3 +, and PD-L2 +CD3 + cells. Panel **(J, K)** & **(L)** show the percentage of PD-1 +CD3 +CD4 +, PD-L1 +CD3 +CD4 +, and PD-L2 +CD3 +CD4 + cells. Panel **(M, N)** & **(O)** show the percentage of PD-1 +CD3 +CD8 +, PD-L1 +CD3 +CD8 +, and PD-L2 +CD3 +CD8 + cells.

In contrast, patients had a higher percentage of PD-L1^+^CD3^+^ cells (3.6 [95% CI 0.3–8.9] vs 1.535 [95% CI 0.7375–2.813]; *P*=0.0109), PD-L1^+^CD3^+^CD8^+^ cells (1.5 [95% CI 0.08–8.78] vs 0.6978 [95% CI 0.0562–1.25]; *P*=0.0065), PD-L2^+^PBMCs (0.46 [95% CI 0.023–2.08] vs 0.0013 [95% CI 0–0.4725]; *P*<0.0001), PD-L2^+^CD3^+^ cells (0.985 [95% CI 0.01–4.2] vs 0.01 [95% CI 0–0.12]; *P*<0.0001) and PD-L2^+^CD3^+^CD4^+^ cells (0.5 [95% CI 0.02–4] vs 0.01 [95% CI 0–1.34]; *P*<0.0001), (Table 1 & Figure 1).

**Table 1:** Percentage of PD-1 / PD-L1 / PD-L2 in immune cells in NSCLC patients and healthy subjects

**Variable**	**N= 10**	**N= 70**	***P***
	**HS**	**NSCLC**	
% of CD3^+^ (T-Lymphocytes)	19.79 ± 5.79	21.21 ± 15.92	0.7813
% CD3^+^CD4^+^ (T-Helper)	61.46 ± 6.36	46.07 ± 14.62	**0.0016**
% CD3^+^CD8^+^ (T-Cytotoxic)	37.67 ± 6.89	35.76 ± 13.53	0.6631
**Variable**	**PD-1**		***P***
	**HS**	**NSCLC**	
% of PBMC	1.154	1.25	0.8103
	(0.8013 - 2.213)	(0.06 - 5.580)	
% of CD3^+^	1.025	1.1	0.9400
(T-Lymphocytes)	(0.725 - 1.45)	(0.11 - 4.5)	
% CD3^+^CD4^+^	0.6113	0.6	0.8550
(T-Helper)	(0.2581 - 0.9688)	(0.013 - 2.6)	
% CD3^+^CD8^+^	0.5645	0.565	0.7881
(T-Cytotoxic)	(0.4038 - 0.7688)	(0 - 3.6)	
**Variable**	**PD-L1**		***P***
	**HS**	**NSCLC**	
% of PBMC	1.141	0.77	**0.0376**
	(0.71 - 2.90)	(0 - 4.62)	
% of CD3^+^	1.535	3.6	**0.0109**
(T-Lymphocytes)	(0.7375 - 2.813)	(0.3 - 8.9)	
% CD3^+^CD4^+^	0.7906	1.1	0.1213
(T-Helper)	(0.2475 - 0.945)	(0.02 - 8.7)	
% CD3^+^CD8^+^	0.6978	1.5	**0.0065**
(T-Cytotoxic)	(0.0562 - 1.25)	(0.08 - 8.78)	
**Variable**	**PD-L2**		***P***
	**HS**	**NSCLC**	
% (PBMC)	0.0013	0.46	**<0.0001**
	(0 - 0.4725)	(0.023 - 2.08)	
% CD3^+^	0.01	0.985	**<0.0001**
(T-Lymphocytes)	(0 - 0.12)	(0.01 - 4.2)	
% CD3^+^CD4^+^	0.01	0.5	**<0.0001**
(T-Helper)	(0 - 1.34)	(0.02 - 4)	
% CD3^+^CD8^+^	0.335	0.625	0.0812
(T-Cytotoxic)	(0 - 1.45)	(0 - 3.06)	

HS= healthy subjects; NSCLC= non-small cell lung cancer, PBMC= peripheral blood mononuclear cells.

### PD-1/PD-L1 & PD-L2 expression status (%) and cytokines levels

In NSCLC patients, the percentage of CD3^+^PD-L1^+^, CD3^+^PD-L2^+^, CD3^+^CD4^+^PD-L1^+^ and CD3^+^CD4^+^PD-L2^+^ cells negatively correlated with the levels of IL-2 (*P*=0.019, *P*=0.044, *P*=0.009 and *P*=0.036; respectively). A negative correlation was also found between IL-6 levels and the percentage of CD3^+^CD4^+^PD-L1^+^, CD3^+^PD-L2^+^ and CD3^+^CD8^+^PD-L2^+^ cells (*P*=0.014, *P*=0.010 and *P*=0.017; respectively). Similarly, the levels of IL-8 negatively correlated with the percentage of CD3^+^PD-L2^+^ and CD3^+^CD4^+^PD-L2^+^ cells (*P*=0.044 and *P*=0.017; respectively). Finally, the plasma concentration of IL-17A negatively correlated with the percentage of CD3^+^CD8^+^PD-L1^+^, CD3^+^CD8^+^PD-L2^+^ and CD3^+^CD4^+^PD-L2^+^ cells (*P*=0.008, *P*=0.018 and *P*=0.025; respectively). In contrast, there was a positive correlation between the concentration of TNF-α and the percentage of CD3^+^PD-L1^+^, CD3^+^PD-L2^+^, CD3^+^CD4^+^PD-L1^+^, CD3^+^CD4^+^PD-L2^+^, CD3^+^CD8^+^PD-L1^+^ and CD3^+^CD8^+^PD-L2^+^ cells (*P*=0.003, *P*=0.003, *P*=0.032, *P*=0.002, *P*=0.003 and *P*=0.028; respectively). Similarly, the concentration of IL-31 positively correlated with the percentage of CD3^+^PD-L2^+^ and CD3^+^CD4^+^PD-L2^+^ (*P*=0.02 and *P*=0.046; respectively), (Supplementary Table 2).

### PD-1/PD-L1 & PD-L2 expression status (%) and clinical characteristics

The percentage of PD-L1^+^CD3^+^CD4^+^ and PD-1^+^CD3^+^CD8^+^ cells positively correlated with age [*P*= 0.028 and *P*=0.049, respectively]. Tobacco exposure was associated with higher percentage of PD-1^+^PBMCs [*P*=0.010]. The percentage of PD-1^+^, PD-L1^+^ and PD-L2^+^ cells was not associated with gender, histology pattern, or clinical stage. EGFR mutation status was associated with a lower percentage of PD-L2^+^CD3^+^ and PD-L2^+^CD3^+^CD8^+^ (*P*=0.051 and *P*=0.008). Decline in functional status (ECOG ≥ 2) was associated with an increase in the percentage of PD-1^+^CD8^+^ cells [95% CI 0.55 – 1.63; *P*= 0.041], (Table 2).

**Table 2:** Bivariate analysis of percentage of expression of PD-1 / PD-L1 /PD-L2 and clinical characteristics of NSCLC patients

Variable	% (PBMC)			% CD3^+^ (T-Lymphocytes)			% CD3^+^CD4^+^ (T-Helper)			% CD3^+^CD8^+^ (T-Cytotoxic)		
	% PD-1	% PDL-1	% PDL-2	% PD-1	% PDL-1	% PDL-2	% PD-1	% PDL-1	% PDL-2	% PD-1	% PDL-1	% PDL-2
Age												
≤60	1.2	0.7	0.45	1.1	3.1	0.98	0.6	0.71	0.42	0.5	1.2	0.45
>60	1.3	0.78	0.46	1.1	3.6	1.1	0.6	1.3	0.6	0.6	2.1	0.78
*P*	0.358	0.72	0.765	0.542	0.231	0.844	0.368	**0.028**	0.272	**0.049**	0.094	0.807
Gender												
Female	1.1	0.69	0.45	1.04	3.55	0.98	0.58	1.1	0.58	0.39	1.3	0.39
Male	1.15	0.85	0.65	1.1	3.6	1.05	0.6	1.1	0.44	0.7	1.9	0.73
*P*	0.101	0.572	0.378	0.352	0.326	0.995	0.141	0.495	0.215	0.062	0.431	0.647
Smoking												
Negative	1	0.67	0.4	0.98	3	0.98	0.64	1.04	0.5	0.39	1.3	0.39
Positive	1.53	1.1	0.69	1.1	4	1.1	0.58	1.4	0.51	0.59	1.85	0.72
*P*	**0.01**	0.069	0.118	0.402	0.185	0.505	0.363	0.135	0.781	0.201	0.319	0.457
Histology												
Adenocarcinoma	1.2	0.77	0.46	1.1	3.8	0.98	0.6	1.1	0.51	0.48	1.55	0.65
Other	1.6	0.7	0.67	1	2.35	1.35	0.42	0.56	0.49	0.74	1.25	0.5
*P*	0.712	0.684	0.839	0.796	0.245	0.705	0.465	0.096	0.719	0.093	0.512	0.314
EGFR status												
Negative	1.3	0.78	0.46	1.1	3.6	1.1	0.6	1.1	0.52	0.57	1.5	0.67
Positive	0.57	0.22	0.34	0.99	2.34	0.37	0.6	1.7	0.33	0.4	1.2	0.08
*P*	0.066	0.197	0.218	0.945	0.568	**0.051**	0.373	0.615	0.24	0.681	0.361	**0.008**
Clinical Stage												
≤IIIB	1.15	0.6	0.72	0.89	2.35	0.94	0.38	0.94	0.44	0.59	1.4	0.62
IV	1.25	0.79	0.46	1.1	3.8	0.99	0.61	1.1	0.51	0.52	1.65	0.63
*P*	0.611	0.375	0.817	0.789	0.471	0.959	0.093	0.853	0.789	0.328	0.567	0.625
ECOG												
1	1.2	0.73	0.46	1.05	3.5	0.98	0.6	1.1	0.5	0.55	1.45	0.58
≥ 2	1.89	1.62	0.74	2.2	7.47	2.25	1.65	3.45	1.27	1.63	5.59	1.75
*P*	0.387	0.093	0.944	0.069	0.075	0.192	0.116	0.259	0.072	**0.041**	0.105	0.153
Metastasis												
Negative	1.35	0.55	0.57	1	3.4	1.9	0.42	1.45	0.6	0.6	1.45	1.4
Positive	1.2	0.79	0.46	1.1	3.8	0.98	0.61	1.1	0.46	0.45	1.55	0.56
*P*	0.913	0.562	0.853	0.608	0.585	0.059	0.338	0.41	0.063	0.182	0.712	0.156
CNS mets												
Negative	1.3	0.76	0.56	0.97	3.4	0.88	0.5	1.1	0.4	0.53	1.4	0.43
Positive	1.1	0.9	0.45	1.2	5.46	1.6	0.67	1.1	0.6	0.7	2.3	0.9
*P*	0.893	0.48	0.215	0.193	0.141	**0.015**	0.206	0.672	**0.015**	0.492	0.19	**0.033**

HS= healthy subjects, NSCLC= non-small cell lung cancer, ECOG= Eastern Cooperative Oncology Group, CNS mets=central nervous system.

### Clinical factors associated with OS

The mean follow-up of patients was 22.9 months, with a range of 18.6 to 27.2 months. The median OS was 17.5 months (95%CI 9.7 - 25.4). Clinical and demographic characteristics (gender, age >60, smoking history, histology, EGFR status, metastases, CNS metastases at diagnosis) did not affect median OS when assessed in a multivariate analysis. Patients with an ECOG ≤1 also had a better OS (20.4 vs. 1.84; *P*=0.08), Supplementary Table 3.

### PD-1/PD-L1 & PD-L2 expression status (%) and OS

**Figure 2:**
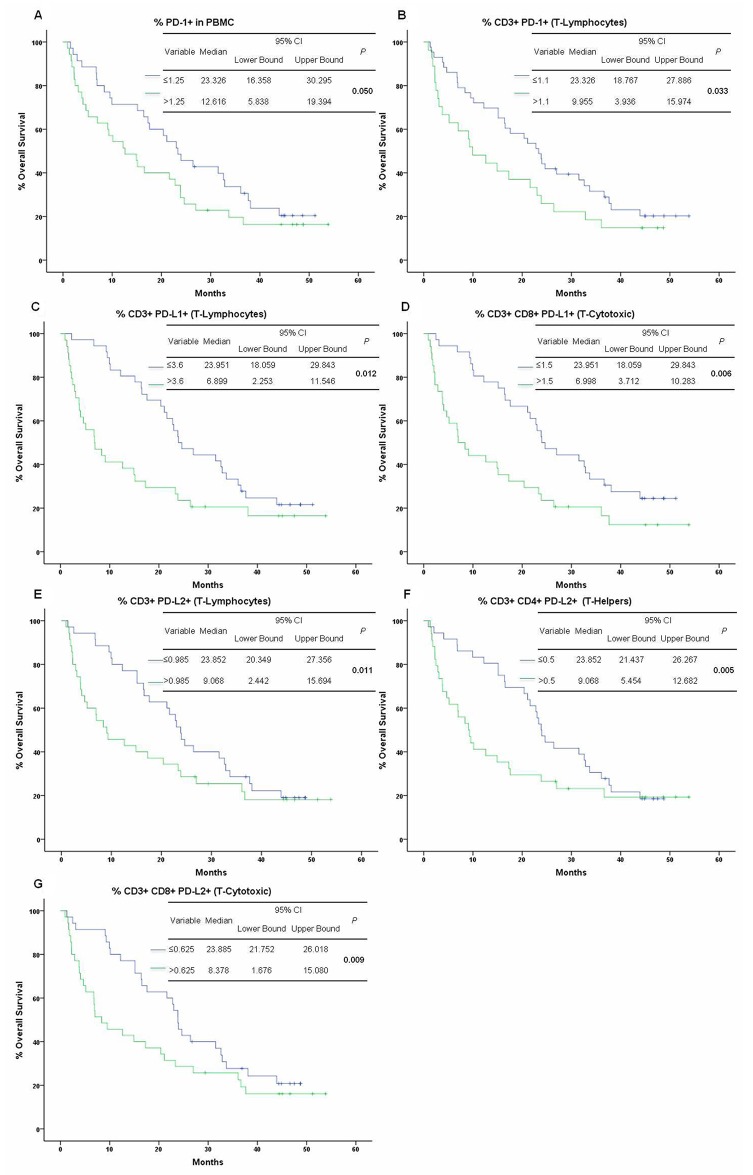
Kaplan-Meier curves of OS by PD-1, PD-L1, PD-L2 expression status **(A)** PD-1 cutoff point ≤1.25% in PBMCs and **(B)** ≤1.1% in CD3 + cells; **(C)** PD-L1 cutoff point ≤3.6% in CD3 + cells and **(D)** ≤1.5% in CD3 +CD8 + cells; **(E)** PD-L2 cutoff point ≤0.985% in CD3 + cells, **(F)** ≤0.5% in CD3 +CD4 + cells, and **(G)** ≤0.625% in CD3 +CD8 + cells.

PD-1 expression on PBMCs and on CD3^+^ cells negatively correlated with OS (*P*=0.050 & *P*=0.033). Patients with >1.25% of PD-1^+^PBMCs had an OS of 12.6 months (95% CI 5.8-19.3) vs patients with ≤1.25% of PD-1^+^PBMCs, whose OS was 23.3 months (95% CI 16.3-30.2). Patients with >1.1% of CD3^+^PD-1^+^ had an OS of 9.9 months (95% CI 3.9-15.9) vs. patients with ≤1.1% of CD3^+^PD-1^+^, whose OS was 23.3 months (95% CI 18.7-27.8), (Figure 2A-2B; Supplementary Table 4). There was a significant reduction in the OS of patients with a high percentage of PD-L1^+^CD3 and PD-L1^+^CD8 cells (*P*=0.012, *P*=0.006, respectively). Patients with >3.6% of CD3^+^PD-L1^+^ or with > 1.5% of CD3^+^CD8^+^PD-L1^+^ cells had an inferior survival rate (6.8 months and 6.9 months) compared with the patients with ≤ these cutoff values (*P*=0.012), (Figure 2C-2D; Supplementary Table 4). No significant associations were found between OS and the percentage of PD-L2^+^PBMCs. In contrast, patients with a high percentage of PD-L2^+^CD3^+^ (>0.985%), PD-L2^+^CD4^+^ (>0.5%) and PD-L2^+^CD3^+^CD8^+^ (>0.625%) cells showed approximately a 15 month decrease in OS (*P*=0.011, *P*=0.005, *P*=0.009), (Figure 2E-2G; Supplementary Table 4).

Calculation of the correlation coefficients for every combination of the predictors selected from the univariate analysis (Supplementary Table 5) revealed a high number of significant correlations, suggesting that collinearity could be a problem for a standard multivariate analysis. Indeed, a collinearity diagnostic test (Supplementary Table 6) yielded tolerances approaching ≅0.1, VIF values >3 and eigen values approaching ≅0, all consistent with a high multicollinearity between several of the predictors. Therefore, it was decided to separate each of the predictors into independent multivariate models that included clinical stage and ECOG only. This analysis (Table 3) shows that NSCLC patients are more likely to have reduced OS if they have a high percentage of PD-1^+^CD3^+^CD4^+^ (HR= 1.695; *P*=.025), PD-L1^+^CD3^+^ (HR=1.248; *P*<0.0001), PD-L1^+^CD3^+^CD8^+^ (HR=1.291; *P*<0.0001) and PD-L2^+^CD3^+^ cells (HR=1.370; *P*=0.011). In contrast to the univariate analysis, in the multivariate analysis a high percentage of PD-1^+^CD3^+^, PD-1^+^PBMCs, PD-L2^+^CD3^+^CD4^+^ and PD-L2^+^CD3^+^CD8^+^ cells was no longer significantly associated with reduced OS.

**Table 3:** Multivariate analysis for prediction of overall survival in NSCLC patients

Model 1. ECOG & clinical Stage					
Variable		HR	95 % Confidence Interval		*P*
			Lower Bound	Upper Bound	
PD-1	% of PBMC	1.234	0.978	1.557	0.077
	% of CD3^+^ (T-Lymphocytes)	1.193	0.855	1.665	0.299
	% CD3^+^CD4^+^ (T-Helper)	1.695	1.068	2.69	**0.025**
	% CD3^+^CD8^+^ (T-Cytotoxic)	1.188	0.776	1.819	0.427
PD-L1	% of PBMC	1.293	0.951	1.757	0.101
	% of CD3^+^ (T-Lymphocytes)	1.248	1.106	1.41	**<0.0001**
	% CD3^+^CD4^+^ (T-Helper)	0.978	0.926	1.033	0.425
	% CD3^+^CD8^+^ (T-Cytotoxic)	1.291	1.118	1.491	**<0.0001**
PD-L2	% (PBMC)	1.122	0.637	1.974	0.69
	% CD3^+^ (T-Lymphocytes)	1.37	1.075	1.745	**0.011**
	% CD3^+^CD4^+^ (T-Helper)	1.009	0.721	1.411	0.96
	% CD3^+^CD8^+^ (T-Cytotoxic)	1.342	0.988	1.823	0.06

HR= hazard ratio, PBMC= peripheral blood mononuclear cells.

## DISCUSSION

The expression of PD-1, PD-L1 & PD-L2 is modulated by the inflammatory milieu, through the action of cytokines [27]. Our results indicate that levels of several cytokines are strongly associated with the proportion of specific T-cell subsets expressing PD-1, PD-L1 & PD-L2. The pleiotropic nature of cytokine signaling make it difficult to determine the exact relationship between cytokine levels and T-cell expression of PD-1, PD-L1 & PD-L2. However, it is worth drawing attention to IL-2 and TNF-α since these cytokines showed the highest number of significant correlations. IL-2 negatively correlated with PD-L1 and PD-L2 expression status (%) in total T-cells (CD3+) and helper T-cells (CD3+CD4+).

It has previously been reported that IL-2 increases PD-L1 expression on human T cells [28], which would be in stark contrast with the negative IL-2 correlation that we found. However, PD-1 activation increases the expression of PTEN while inhibiting the PI3K and Akt pathways [29], which can lead to a decrease in the synthesis and release of IL-2 by T-cells. Indeed, it has been shown that blocking the PD-1/PD-L1 axis increases IL-2 production by T-cells without stimulating their proliferation [30]. Whereas most correlations were negative, TNF-α and IL-31 were the only two cytokines showing a positive association with T-cell subsets. Plasma levels of TNF-α positively correlated with PD-L1 and PD-L2 expression status (%) in total T-cells (CD3+), helper T-cells (CD3+CD4+) and cytotoxic T-cells (CD3+CD8+). This finding is in agreement with mechanistic studies showing that TNF-α induces PD-L1 expression on the surface of T cells and monocytes [10, 27, 31]. Our results indicate that TNF-α might have a similar effect on PD-L2 expression.

The efficacy of PD-1/PD-L1 ICIs has been demonstrated in numerous preclinical models [29, 32] as well as in several human clinical trials [33–36]. Although PD-1 blockade has dramatically improved the response rate of NSCLC patients, the identification of biomarkers predictive of response has remained elusive. PD-L1 expression in tumors has been evaluated in several studies but its prognostic and predictive value continue to be a matter of debate [37].

It is likely that some of the reliability and reproducibility issues surrounding the use of PD-L1 as a biomarker derive from variable assay methodology, heterogeneity of tumor sampling and surgical inaccessibility [38]. Novel studies have hypothesized that PD-L1 expression in CTCs, found in malignant pleural effusion or in blood from patients with metastatic NSCLC, represents an accurate surrogate for the determination of tumor PD-L1 levels in malignant cells of the primary tumor [24]. However, it has also been suggested that nivolumab may exert its effect on PD-L1 negative patients by a mechanism that is independent of tumoral PD-L1 expression, for example by blocking other inhibitory ligands of the PD-1 receptor such as PD-L2 and thus decreasing the inhibition of T-cells [21].

Our results concur that there are mechanisms independent of tumoral PD-L1 expression by which tumor evasion occurs. Although no differences were found between patients and controls with regards to the percentage of peripheral PBMCs, CD3^+^, CD3^+^CD4^+^ and CD3^+^CD8^+^ cells, NSCLC patients had a higher percentage of circulating PD-L1^+^CD3^+^ and PD-L1^+^CD3^+^CD8^+^ cells (but not PD-L1^+^CD3^+^CD4^+^), as well as a higher percentage of PD-L2^+^PBMCs, PD-L2^+^CD3^+^ and PD-L2^+^CD3^+^CD4^+^ cells (but not PD-L2^+^CD3^+^CD8^+^).

Furthermore, the survival analysis revealed a dramatic reduction in the OS of patients with a high percentage of PD-1^+^PBMCs, PD-1^+^CD3^+^, PD-L1^+^CD3^+^, PD-L1^+^CD3^+^CD8^+^, PD-L2^+^CD3^+^, PD-L2^+^CD3^+^CD4^+^ and PD-L2^+^CD3^+^CD8^+^ cells. However, in the multivariate analysis only a high percentage of PD-1^+^CD3^+^CD4^+^, PD-L1^+^CD3^+^, PD-L1^+^CD3^+^CD8^+^ and PD-L2^+^CD3^+^ cells were significantly associated with reduced OS in NSCLC patients. This is likely due to the tight interrelationship governing the expression of receptor and ligands from the same signaling pathway, which causes multicollinearity problems that reduce the power of a multivariate analysis. It is possible that a larger scale study or a different regression model would confirm that, in addition to the predictors previously described, a high percentage of PD-1^+^CD3^+^, PD-1^+^PBMCs, PD-L2^+^CD3^+^CD4^+^ and PD-L2^+^CD3^+^CD8^+^ cells would also be associated with reduced OS in NSCLC patients.

Nevertheless, our results indicate that an additional source of PD-1 inhibitory ligands are T-cells themselves. This could represent an additional and important mechanism by which tumor immune evasion occurs in advanced NSCLC patients. However, future studies are necessary to provide a mechanistic insight into how the expression of these proteins leads to tumor immune escape.

This study produced results which corroborate and expand on the findings of previous work in this field. For instance, Zhang *et al.* reported a significant difference between the mean levels of PD-L1 in the blood serum of advanced NSCLC patients and healthy controls, being 0.723 (±0.081) ng/ml and 0.565 (±0.048ng/ml), respectively. By establishing a cut-off point of 0.636 ng/ml, the authors distinguished a clear correlation with survival and found an area under the curve of 0.956 (95%CI 0.927–0.985) [41]. A more recent study by Meniawy *et al.* (2016) found that: a) NSCLC patients have a higher percentage of PD-L1^+^CD3^+^ and PD-L1^+^CD3^+^CD8^+^ cells; b) reduced OS is significantly associated (in a univariate analysis) with a high percentage of PD-L1^+^CD3^+^ and PD-L1^+^CD3^+^CD8^+^ cells; c) reduced OS is significantly associated (in a multivariate analysis) with a high percentage of PD-L1^+^CD3^+^ cells [42]. However, in the current study no differences were found between patients and healthy subjects with regards to the percentage of PD-L1^+^CD3^+^CD4 cells, nor were there any associations found between EGFR mutation status and PD-L1 expression. On the other hand, we found that patients with EGFR mutations had a lower percentage of PD-L2^+^CD3^+^ and PD-L2^+^CD3^+^CD8^+^ cells, which to the best of our knowledge had not previously been described. Finally, our multivariate analysis revealed that a high percentage of PD-1^+^CD3^+^CD4^+^, PD-L1^+^CD3^+^, PD-L1^+^CD3^+^CD8^+^ and PD-L2^+^CD3^+^ cells is significantly associated with reduced OS. These minor discrepancies could be attributed to differences in the baseline characteristics of patients. For instance, whereas our patients were treatment-naïve at the time of sample collection, the majority of patients included in the study by Meniawy et al (2016) had received at least one line of systemic therapy (chemotherapy). Nevertheless, the results from both studies highlight the prognostic value of assessing the expression of PD-1 and its ligands on the surface of peripheral T-cells. In conclusion, it is likely that patients with increased expression of PD-L1 and PD-L2 on the surface of T-cells could benefit from PD-1/PD-L1/PD-L2 blockade even if their tumors are negative for these molecules. This would certainly explain why a significant number of patients with PD-L1 negative tumors have shown responses to anti-PD-1and anti-PD-L1 ICIs [39, 40]

Our data highlight the need for future studies evaluating the efficacy of anti-PD-1 and anti-PD-L1 therapies in patients with differential expression of PD-L1 and PD-L2 on peripheral T-lymphocytes. This may represent a potential biomarker to noninvasively predict the therapeutic efficacy of PD-1/PD-L1 blockade in patients

## MATERIALS AND METHODS

### Study design

In this prospective observational study, a total of 70 treatment-naïve patients with advanced NSCLC (stage IIIB & IV) were recruited from May 2013 to June 2014 at the Lung Cancer Clinic of the “Instituto Nacional de Cancerología” of Mexico (INCan). The inclusion criteria were: Adult patients (>18 years), newly diagnosed, histopathological confirmation of NSCLC, disease stage IIIB or IV, Eastern Cooperative Oncology Group (ECOG) status of 0–2, no history of autoimmune diseases or of recent steroid therapy, without prior treatment (radiation, chemotherapy or immunotherapy) but eligible to receive standard of care chemotherapy (Platinum-Taxol, Pemetrexed, Platinum-Gemcitabine and Platinum Pemetrexed) or TKIs at the time of diagnosis. Clinicopathological characteristics were recorded from patients at the time of diagnosis. Blood samples of healthy subjects (N=10), paired by age and gender, and with complete information regarding smoking history, wood-smoke exposure and comorbidities, were obtained from the Blood Transfusion Center bank. Written informed consent was obtained from each patient prior to enrolment. This protocol was approved by the Institutional Review Board and Ethics Committee (INCAN [011/018/ICI] [CB/683]) of the INCan. This study is registered with ClinicalTrials.gov (NCT02758314).

### Sample collection

Blood samples were prospectively collected (pretreatment) and handled as follows: a) Eight milliliters of blood were drawn into a plastic EDTA tube (BD Biosciences; San Jose, Ca, US). Upon collection, the plasma was separated by centrifugation and stored at -80°C for subsequent cytokine analysis; b) Eight milliliters of blood were drawn into 3.2% citrate tubes (BD Biosciences). Upon collections samples were diluted with PBS (1:1, v:v) and separated by density gradient centrifugation (Lymphoprep; AXIS-SHIELD PoC AS, Norway). The interphase, containing peripheral blood mononuclear cells (PBMCs), was stored in liquid nitrogen for subsequent immunophenotyping.

### Immunophenotyping

Expression of PD-1/PD-L1/PD-L2 was assessed retrospectively in prospectively collected blood samples. The following combination of human monoclonal antibodies were used according to the manufacturers’ instructions to identify different immune cell populations (CD3^+^, CD4^+^ and CD8^+^ T lymphocytes): anti-CD3-FITC, anti-PD-L1-PE, anti-CD8-APC Cy7, anti-PD-1-PE Cy7, anti-PD-L2-APC and anti-CD4-PerCp (BioLegend; San Diego, CA, US). The PBMC fraction was blocked for 5 minutes with a FAB anti-IgG. Samples were incubated for 45 minutes with the appropriate antibodies (2.5 μl) at room temperature and protected from light exposure. After incubation, 2 ml of a 1:1 solution of PBS-Fetal bovine serum (FBS) were added to each sample. Samples were then centrifuged at 1100 rpm for 5 minutes. The supernatant was decanted and cells were fixed in paraformaldehyde (1%).

Gating strategy were set using fluorescence minus one (FMO) for PD-1 /PD-L1 &PD-L2. The samples were acquired in a FACS Aria II Flow Cytometer (BD, Biosciences, San José, Cal, USA) and analyzed with FlowJo software 10.1 (Tree Star. Ashland, Or, USA). The leukocyte population was gated based on morphological parameters on a forward vs side scatter (FSC/SSC) plot.

### Measurement of cytokines and chemokines

Plasma levels of 14 cytokines (IL-1β, IL-2, IL-4, IL-6, IL-8, IL-10, IL-12 p70, IL-17A, IL-27, IL-31, IL-29, and IL-33, TNF-α, IFN-γ) were quantified as previously described [26]. Briefly, a Pro-Inflammatory and Th1/Th2/Th17 cytometric bead array assay kit (BD, San Jose, CA, USA) was used according to the manufacturer's instructions. Events were acquired using a FACS Aria II Flow Cytometer (BD, Biosciences, Mexico) and analyzed with FCAP Array Software V. 3.0 (Soft Flow, Pecs, Hungary).

### Statistical analysis

Continuous data were summarized as arithmetic means with standard deviations (SDs) or medians with ranges according to data distribution. Two group comparisons were tested using Student's t test or Mann-Whitney U (according to data distribution determined by the Kolmogorov Smirnov test). Nominal data was analyzed using the chi square (X^2^) test. Immune parameters associated with clinical variables were determined by bivariate analysis. Correlations were computed by linear regression and analyzed using the Spearman rank correlation test. The Receiver Operating Characteristic (ROC) method was performed to find the best cut-off point value for PD-1, PD-L1 and PD-L2 to be used for the survival analysis. OS curves were estimated by the Kaplan–Meier method while comparisons among groups were analyzed with log-rank or Breslow tests. Statistically significant and borderline variables (*P* values ≤0.1) were included in the multivariate analyses. Statistical significance was determined as *P* ≤0.05 with a two-sided test. All data were analyzed using the SPSS software package version 20 (SPSS, Inc., Chicago, IL, US).

## SUPPLEMENTARY MATERIALS FIGURES AND TABLES





## Abbreviations

EGFR: Epidermal Growth Factor Receptor
HS: Healthy subject(s)
ICIs: Immune checkpoint inhibitor
NSCLC: Non-small cell lung cancer
PD-1: Programmed Death-1 receptor
PD-L1: Programmed Death Ligand-1
PD-L2: Programmed Death Ligand-2
PBMC: Peripheral Blood Mononuclear Cell
TKI: Tyrosine Kinase Inhibitor
Treg: Regulatory T-cell(s)
Th: Helper T-cell(Th)
SD: Standard Deviation
