# Supplementary material for: Expression of PD-1/PD-L1 and PD-L2 in peripheral T-cells from non-small cell lung cancer patients
Source: Oncotarget. 2017 Oct 24;8(60):101994–2005. doi: 10.18632/oncotarget.22025 (PMC5731930; doi:10.18632/oncotarget.22025)
Supplement: Supplementary file 1 [file oncotarget-08-101994-s001.pdf]

Expression of PD-1/PD-L1 and PD-L2 in peripheral T-cells from non-small cell lung cancer patients

SUPPLEMENTARY MATERIALS

Supplementary Table 1: Baseline clinical characteristics of NSCLC patients and healthy subjects

Variable	HS N=10	NSCLC N=70	*P*
Age			
Median (range)	59 (33 - 74)	63 (35 - 90)	
≤60	60 (6/10)	41.4 (29/70)	0.32
>60	40 (4/10)	58.6 (41/70)	
Gender			
Female	40 (4/10)	48.6 (34/70)	0.741
Male	60 (6/10)	51.4 (36/70)	
Smoking			
Negative	100 (10/10)	45.7 (32/70)	0.001
Positive	0 (0/10)	54.3 (38/70)	
Wood Smoke			
Negative	90 (9/10)	68.6 (48/70)	0.267
Positive	10 (1/10)	31.4 (22/70)	
Diabetes mellitus			
Negative	70 (7/10)	82.9 (58/70)	0.387
Positive	30 (3/10)	17.1 (12/70)	
Hypertension			
Negative	70 (7/10)	62.9 (44/70)	0.74
Positive	30 (3/10)	37.1 (26/70)	
Histology			
Adenocarcinoma		88.6 (62/70)	
Other		11.4 (8/70)	
EGFR status			
Negative		67.1 (47/70)	
Del 19		25.7 (18/70)	
L858R		7.1 (5/70)	
Clinical Stage			
≤IIIB		14.3 (10/70)	
IV		85.7 (60/70)	
ECOG			
1		97.1 (68/70)	
≥2		2.9 (2/70)	
Metastases			
Negative		14.3 (10/70)	
Positive		85.7 (60/70)	
CNS mets			
Negative		64.3 (45/70)	
Positive		35.7 (25/70)	
Pleural Effusion			
Negative		31.4 (22/70)	
Positive		68.6 (48/70)	
1º Line Treatment			
Platinum + Taxol		80 (56/70)	
TKI		7.1 (5/70)	
Pemetrexed		4.3 (3/70)	
Platinum + Gemcitabine		2.9 (2/70)	
Platinum + Pemetrexed		5.7 (4/70)	

HS= healthy subjects; NSCLC= non-small cell lung cancer, ECOG= Eastern Cooperative Oncology Group, CNS mets=central nervous system metastases.

Supplementary Table 2: Correlation analysis of Immune cell subsets and cytokines in NSCLC patients

Variable		IL-17A	IFN-γ	IL-4	IL-2	IL-1β	IL-6	IL-8	IL-10	IL-12p70	TNF-α	IL-27	IL-29	IL-31	IL-33
% of PD-1^{+}	rs	0.134	0.194	0.249	-0.029	-0.171	0.156	0.044	0.210	-0.064	0.194	0.129	-0.015	0.031	-0.017
in PBMC	*P*	0.352	0.176	0.082	0.840	0.236	0.280	0.759	0.144	0.657	0.178	0.371	0.920	0.832	0.908
% of CD3^{+}PD-1^{+}	rs	0.221	0.126	-0.002	0.271	-0.078	0.031	0.220	0.047	-0.060	0.178	0.197	-0.111	0.117	-0.044
(T-Lymphocytes)	*P*	0.123	0.384	0.988	0.057	0.591	0.831	0.125	0.745	0.679	0.217	0.170	0.441	0.417	0.762
% of CD3^{+}CD4^{+}PD-1^{+}	rs	0.188	0.246	0.064	0.194	-0.244	0.164	0.192	0.006	0.051	0.051	0.040	0.029	0.021	-0.229
(T-Helper)	*P*	0.192	0.085	0.659	0.176	0.088	0.256	0.181	0.969	0.726	0.727	0.784	0.839	0.883	0.110
% of CD3^{+}CD8^{+}PD-1^{+}	rs	0.150	-0.057	-0.083	-0.054	-0.109	-0.094	0.054	-0.001	0.108	0.156	0.097	-0.025	-0.056	-0.089
(T-Cytotoxic)	*P*	0.299	0.693	0.567	0.712	0.453	0.516	0.711	0.993	0.456	0.278	0.501	0.863	0.699	0.538
% of PD-L1^{+}	rs	0.140	0.107	0.256	-0.083	-0.15	0.121	0.021	0.220	-0.112	0.226	0.134	-0.086	-0.006	0.015
in PBMC	*P*	0.334	0.457	0.073	0.565	0.299	0.405	0.888	0.125	0.437	0.114	0.355	0.555	0.967	0.920
% of CD3^{+}PD-L1^{+}	rs	-0.221	-0.066	-0.185	**-.331***	-0.145	-0.139	-0.039	0.166	-0.065	**.418****	0.132	-0.102	0.187	-0.017
(T-Lymphocytes)	*P*	0.123	0.650	0.199	**0.019**	0.315	0.334	0.789	0.249	0.653	**0.003**	0.360	0.481	0.193	0.906
% of CD3^{+}CD4^{+}PD-L1^{+}	rs	-0.123	-0.178	-0.264	**-.366****	-0.060	**-.344***	-0.143	0.136	-0.114	**.304***	-0.187	-0.258	0.129	0.008
(T-Helper)	*P*	0.396	0.217	0.064	**0.009**	0.680	**0.014**	0.323	0.347	0.429	**0.032**	0.192	0.071	0.372	0.954
% of CD3^{+}CD8^{+}PD-L1^{+}	rs	**-.369****	-0.109	-0.127	-0.242	-0.076	-0.249	-0.110	0.112	-0.085	**.410****	0.223	0.001	0.188	-0.040
(T-Cytotoxic)	*P*	**0.008**	0.452	0.379	0.091	0.599	0.081	0.447	0.439	0.558	**0.003**	0.120	0.997	0.190	0.785
% of PD-L2^{+}	rs	-0.127	0.008	0.089	-0.192	-0.197	0.081	-0.006	0.078	-0.017	0.065	-0.017	0.047	0.071	-0.173
in PBMC	*P*	0.381	0.958	0.538	0.181	0.171	0.574	0.970	0.589	0.905	0.654	0.904	0.747	0.624	0.229
% of CD3^{+}PD-L2^{+}	rs	-0.249	0.012	-0.237	**-.287***	0.011	**-.361***	**-.287***	0.159	-0.078	**.410****	0.002	0.184	**.327***	0.023
(T-Lymphocytes)	*P*	0.081	0.936	0.097	**0.044**	0.940	**0.010**	**0.044**	0.269	0.591	**0.003**	0.991	0.200	**0.020**	0.873
% of CD3^{+}CD4^{+}PD-L2^{+}	rs	**-.316***	-0.123	-0.122	**-.297***	-0.057	-0.272	**-.337***	0.154	0.042	**.424****	0.113	-0.09	**.283***	-0.017
(T-Helper)	*P*	**0.025**	0.394	0.400	**0.036**	0.692	0.056	**0.017**	0.286	0.772	**0.002**	0.436	0.534	**0.046**	0.906
% of CD3^{+}CD8^{+}PD-L2^{+}	rs	**-.334***	-0.087	-0.216	-0.141	-0.079	**-.337***	-0.259	0.093	0.059	**.311***	0.043	0.236	0.200	-0.026
(T-Cytotoxic)	*P*	**0.018**	0.55	0.131	0.328	0.584	**0.017**	0.069	0.519	0.684	**0.028**	0.767	0.099	0.164	0.856

rs= Spearman correlation coefficient. PBMC= peripheral blood mononuclear cells.

Supplementary Table 3: Clinical associated factors with overall survival

Variable	Median	95% Confidence interval		*P*
		Lower bound	Upper bound	
OS	17.577	9.7331	25.423	
Age (years)				
≤60	17.281	8.155	26.408	0.438
>60	20.402	11.025	29.78	
Gender				
Female	22.998	13.564	32.432	0.124
Male	15.146	0	30.48	
Smoking history				
Negative	20.402	11.023	29.782	0.204
Positive	15.146	5.817	24.474	
Histology				
Adenocarcinoma	16.427	8.251	24.603	0.155
Others	23.885	16.417	31.353	
EGFR status				
Negative	16.559	10.366	22.751	0.229
Positive	32.854	22.81	42.899	
Clinical Stage				
IIIB	32.624	NR	NR	**0.033**
IV	16.427	8.917	23.938	
Metastases				
Negative	27.006	13.465	40.548	0.104
Positive	16.559	8.115	25.002	
ECOG				
1	20.402	13.765	27.04	0.08
2	1.84	NR	NR	
CNS Mets				
Negative	21.092	12.887	29.298	0.458
Positive	14.916	2.262	27.57	

ECOG= Eastern Cooperative Oncology Group; CNS Mets=central nervous system metastases.

Supplementary Table 4: Immune cells populations associated with overall survival

Variable	Median	95% Confidence interval		*P*
		Lower bound	Upper bound	
% PD-1$^+$ in PBMC				
≤1.25	23.326	16.358	30.295	**0.050**
>1.25	12.616	5.838	19.394	
% of CD3$^+$PD-1$^+$ (T-Lymphocytes)				
≤1.1	23.326	18.767	27.886	**0.033**
>1.1	9.955	3.936	15.974	
% of CD3$^+$CD4$^+$PD-1$^+$ (T-Helper)				
≤0.6	23.326	19.01	27.643	0.071
>0.6	12.189	4.403	19.975	
% of CD3$^+$CD8$^+$PD-1$^+$ (T-Cytotoxic)				
≤0.565	21.092	12.372	29.813	0.401
>0.565	17.577	4.058	31.096	
% of PD-L1$^+$ in PBMC				
≤1.77	22.998	7.064	38.932	0.122
>1.77	14.916	7.604	22.227	
% of CD3$^+$PD-L1$^+$ (T-Lymphocytes)				
≤3.6	23.951	18.059	29.843	**0.012**
>3.6	6.899	2.253	11.546	
% of CD3$^+$CD4$^+$PD-L1$^+$ (T-Helper)				
≤1.1	21.092	12.54	29.645	0.227
>1.1	14.916	0.587	29.244	
% of CD3$^+$CD8$^+$PD-L1$^+$ (T-Cytotoxic)				
≤1.5	23.951	18.059	29.843	**0.006**
>1.5	6.998	3.712	10.283	
% of PD-L2$^+$ in PBMC				
≤2.46	20.402	11.999	28.806	0.620
>2.46	15.146	2.286	28.006	
% CD3$^+$PD-L2$^+$ (T-Lymphocytes)				
≤0.985	23.852	20.349	27.356	**0.011**
>0.985	9.068	2.442	15.694	
% CD3$^+$ CD4$^+$ PD-L2$^+$ (T-Helper)				
≤0.5	23.852	21.437	26.267	**0.005**
>0.5	9.068	5.454	12.682	
% CD3$^+$ CD8$^+$ PD-L2$^+$ (T-Cytotoxic)				
≤0.625	23.885	21.752	26.018	**0.009**
>0.625	8.378	1.676	15.080	

PBMC= peripheral blood mononuclear cells.

Supplementary Table 5 : Correlation analysis of PD-1 /PD-L1 & PD-L2 expression in NSCLC patients

See Supplementary File 1

Supplementary Table 6: Collinearity diagnostic

	Variable	Unstandardized coefficients		Standardized coefficients	t	*P*	Collinearity statistics		Eigenvalue	Condition index
		B	Std. error	Beta			Tolerance	VIF		
	Clinical Stage	1.956	.097		20.063	.000			9.125	1.000
PD-1	% of PBMC	-.078	.087	-.261	-.889	.378	.151	6.612	.028	17.957
	% of CD3+(T-Lymphocytes)	-.079	.075	-.211	-1.049	.299	.321	3.114	.286	5.645
	% CD3+CD4+(T-Helper)	.240	.090	.430	2.668	.010	.499	2.004	.472	4.395
	% CD3+CD8+(T-Cytotoxic)	-.150	.068	-.346	-2.187	.033	.517	1.933	.512	4.220
PD-L1	% of PBMC	.154	.097	.386	1.590	.117	.220	4.546	.062	12.169
	% of CD3+(T-Lymphocytes)	-.017	.031	-.137	-.539	.592	.200	4.999	.039	15.228
	% CD3+CD4+(T-Helper)	.003	.006	.066	.467	.643	.658	1.519	.706	3.596
	% CD3+CD8+(T-Cytotoxic)	.061	.034	.430	1.814	.075	.231	4.335	.139	8.090
PD-L2	% of PBMC	-.076	.112	-.117	-.685	.496	.442	2.263	.342	5.167
	% of CD3+(T-Lymphocytes)	-.050	.074	-.163	-.679	.500	.225	4.435	.090	10.072
	% CD3+CD4+(T-Helper)	.045	.057	.098	.793	.431	.841	1.190	.984	3.046
	% CD3+CD8+(T-Cytotoxic)	-.063	.076	-.171	-.829	.411	.305	3.284	.214	6.528
