# Supplementary material for: Expression of PD-1/PD-L1 and PD-L2 in peripheral T-cells from non-small cell lung cancer patients
Source: Oncotarget. 2017 Oct 24;8(60):101994–2005. doi: 10.18632/oncotarget.22025 (PMC5731930; doi:10.18632/oncotarget.22025)
Supplement: Supplementary file 2 [file oncotarget-08-101994-s002.docx]

**Supplementary Table 5:** Correlation analysis of PD-1 /PD-L1 & PD-L2 expression in NSCLC patients.

| Variable | |  | **PD-1** | | | | **PD-L1** | | | | **PD-L2** | | | |
| --- | --- | --- | --- | --- | --- | --- | --- | --- | --- | --- | --- | --- | --- | --- |
|  |  |  | % of PBMC | % of CD3^+^ | % CD3^+^CD4^+^ | % CD3^+^CD8^+^ | % of PBMC | % of CD3^+^ | % CD3^+^CD4^+^ | % CD3^+^CD8^+^ | % of PBMC | % of CD3^+^ | % CD3^+^CD4^+^ | % CD3^+^CD8^+^ |
|  |  |  |  | (T-Lymphocytes) | (T-Helper) | (T-Cytotoxic) |  | (T-Lymphocytes) | (T-Helper) | (T-Cytotoxic) |  | (T-Lymphocytes) | (T-Helper) | (T-Cytotoxic) |
| **PD-1** | % of PBMC | rs | 1.000 |  |  |  |  |  |  |  |  |  |  |  |
|  |  | P |  |  |  |  |  |  |  |  |  |  |  |  |
|  | % of CD3^+^ | rs | **.371^**^** | 1.000 |  |  |  |  |  |  |  |  |  |  |
|  | (T-Lymphocytes) | P | **.002** |  |  |  |  |  |  |  |  |  |  |  |
|  | % CD3^+^CD4^+^ | rs | **.325^**^** | **.720^**^** | 1.000 |  |  |  |  |  |  |  |  |  |
|  | (T-Helper) | P | **.006** | **.000** |  |  |  |  |  |  |  |  |  |  |
|  | % CD3^+^CD8^+^ | rs | **.267^*^** | **.633^**^** | **.505^**^** | 1.000 |  |  |  |  |  |  |  |  |
|  | (T-Cytotoxic) | P | **.026** | **.000** | **.000** |  |  |  |  |  |  |  |  |  |
| **PD-L1** | % of PBMC | rs | **.864^**^** | **.353^**^** | **.323^**^** | **.277^*^** | 1.000 |  |  |  |  |  |  |  |
|  |  | P | **.000** | **.003** | **.006** | **.020** |  |  |  |  |  |  |  |  |
|  | % of CD3^+^ | rs | **.312^**^** | **.286^*^** | **.253^*^** | **.299^*^** | **.302^*^** | 1.000 |  |  |  |  |  |  |
|  | (T-Lymphocytes) | P | **.008** | **.016** | **.034** | **.012** | **.011** |  |  |  |  |  |  |  |
|  | % CD3^+^CD4^+^ | rs | .066 | .151 | .108 | **.255^*^** | .126 | **.634^**^** | 1.000 |  |  |  |  |  |
|  | (T-Helper) | P | .586 | .211 | .375 | **.033** | .299 | **.000** |  |  |  |  |  |  |
|  | % CD3^+^CD8^+^ | rs | **.227** | **.269^*^** | .190 | **.295^*^** | .217 | **.885^**^** | **.472^**^** | 1.000 |  |  |  |  |
|  | (T-Cytotoxic) | P | **.058** | **.025** | .116 | **.013** | .071 | **.000** | **.000** |  |  |  |  |  |
| **PD-L2** | % of PBMC | rs | **.622^**^** | .006 | -.018 | .041 | **.398^**^** | .149 | .018 | .099 | 1.000 |  |  |  |
|  |  | P | **.000** | .963 | .881 | .738 | **.001** | .220 | .883 | .413 |  |  |  |  |
|  | % of CD3^+^ | rs | .190 | **.262^*^** | **.288^*^** | **.246^*^** | .180 | **.651^**^** | **.346^**^** | **.679^**^** | -.045 | 1.000 |  |  |
|  | (T-Lymphocytes) | P | .114 | **.028** | **.016** | **.040** | .136 | **.000** | **.003** | **.000** | .712 |  |  |  |
|  | % CD3^+^CD4^+^ | rs | .159 | **.252^*^** | **.247^*^** | **.309^**^** | .096 | **.474^**^** | **.345^**^** | **.434^**^** | .062 | **.627^**^** | 1.000 |  |
|  | (T-Helper) | P | .188 | **.036** | **.039** | **.009** | .431 | **.000** | **.003** | **.000** | .607 | **.000** |  |  |
|  | % CD3^+^CD8^+^ | rs | -.006 | .088 | **.262^*^** | .203 | .064 | **.421^**^** | .175 | **.545^**^** | -.164 | **.739^**^** | **.408^**^** | 1.000 |
|  | (T-Cytotoxic) | P | .960 | .469 | **.028** | .092 | .601 | **.000** | .148 | **.000** | .175 | **.000** | **.000** |  |
